# Detection of *KRAS* mutations in plasma cell-free DNA of colorectal cancer patients and comparison with cancer panel data for tissue samples of the same cancers

**DOI:** 10.5808/GI.2019.17.4.e42

**Published:** 2019-11-29

**Authors:** Suji Min, Sun Shin, Yeun-Jun Chung

**Affiliations:** 1 Department of Microbiology, College of Medicine, The Catholic University of Korea, Seoul 06591, Korea; 2 Precision Medicine Research Center, College of Medicine, The Catholic University of Korea, Seoul 06591, Korea; 3 Integrated Research Center for Genome Polymorphism, College of Medicine, The Catholic University of Korea, Seoul 06591, Korea; 4 Cancer Evolution Research Center, College of Medicine, The Catholic University of Korea, Seoul 06591, Korea

**Keywords:** cell-free DNA, digital PCR, *KRAS*, liquid biopsy, next-generation sequencing

## Abstract

Robust identification of genetic alterations is important for the diagnosis and subsequent treatment of tumors. Screening for genetic alterations using tumor tissue samples may lead to biased interpretations because of the heterogeneous nature of the tumor mass. Liquid biopsy has been suggested as an attractive tool for the non-invasive follow-up of cancer treatment outcomes. In this study, we aimed to verify whether the mutations identified in primary tumor tissue samples could be consistently detected in plasma cell–free DNA (cfDNA) by digital polymerase chain reaction (dPCR). We first examined the genetic alteration profiles of three colorectal cancer (CRC) tissue samples by targeted next-generation sequencing (NGS) and identified 11 non-silent amino acid changes across six cancer-related genes (*APC, KRAS, TP53, TERT, ARIDIA*, and *BRCA1*). All three samples had *KRAS* mutations (G12V, G12C, and G13D), which were well-known driver events. Therefore, we examined the *KRAS* mutations by dPCR. When we examined the three *KRAS* mutations by dPCR using tumor tissue samples, all of them were consistently detected and the variant allele frequencies (VAFs) of the mutations were almost identical between targeted NGS and dPCR. When we examined the *KRAS* mutations using the plasma cfDNA of the three CRC patients by dPCR, all three mutations were consistently identified. However, the VAFs were lower (range, 0.166% to 2.638%) than those obtained using the CRC tissue samples. In conclusion, we confirmed that the *KRAS* mutations identified from CRC tumor tissue samples were consistently detected in the plasma cfDNA of the three CRC patients by dPCR.

## Introduction

Cancer is made up of highly heterogeneous cells [1]. In cancer treatment, a major obstacle is posed by the recurrence and metastasis of primary tumor cells after treatment, which are caused by the acquisition of genetic alterations in some tumor cell clones. Therefore, identification of the genetic alteration profile is important for the diagnosis and subsequent treatment of tumors. Currently, genetic alteration profiling is mostly performed using tumor tissues from surgical resection or biopsy samples, which may cause a biased interpretation because those samples correspond to only a part of the heterogeneous tumor mass. Another limitation of tissue-based mutation analysis is that repetitive serial sampling, which is important for the follow-up of treatment outcomes, is almost impossible. Recently, genetic alteration profiling using cell-free nucleic acids has been suggested to overcome the limitations of the tissue-based approach [2,3].

Liquid biopsy refers to the analysis of molecular profiles using bodily fluids rather than solid biological tissues [2,3]. As apoptotic tumor cells are degraded, the non-absorbed intracellular organelles—including cell-free nucleic acids—can be released into the bloodstream. Indeed, cell-free nucleic acids in the blood provide a less biased reflection of the genetic alteration profiles of heterogeneous tumor masses than tumor tissue samples [2,3]. Therefore, mutation analysis using plasma cell–free DNA (cfDNA) is becoming popular for diverse cancers [2-4].

However, cfDNA is usually fragmented and the amount of cfDNA in the blood varies dramatically from patient to patient [2-4]. In addition, the cfDNA released from normal blood cells is much more abundant than the cfDNA from tumor cells [2,3]. Therefore, it is challenging to identify the mutation profiles of tumor cfDNA precisely. However, the development of next-generation sequencing (NGS) has facilitated precise mutation analysis, especially in the field of precision cancer medicine. Nonetheless, although several pieces of evidence have suggested that deep targeted sequencing can detect low-level mutations precisely, the clinical translation of NGS-based mutation profiling using cfDNA still needs more evidence regarding its sensitivity and specificity.

Recently, digital polymerase chain reaction (dPCR) has taken center stage for the ultra-sensitive detection of genetic alterations from a minute amount of nucleic acids [5,6]. Since the identification of mutation profiles from plasma cfDNA must be extremely sensitive and precise, dPCR is ideal for this application. Indeed, several driver alterations such as *EGFR, BRAF*, and *HER2* amplification were successfully identified by dPCR using circulating tumor DNA [7-9]. However, for clinical applications, more evidence will be required to verify the consistency of mutation profiles between plasma cfDNA and original tumor tissue, as well as their consistency with the results of NGS analysis.

In this study, we aimed to develop a dPCR system for detecting *KRAS* mutations using plasma cfDNA from colorectal cancer (CRC) patients and to compare the results with NGS analysis.

## Methods

### CRC tissue samples and tumor DNA extraction

CRC tissue and blood samples were collected from three patients at Seoul St. Mary’s Hospital (Seoul, Korea) with Institutional Review Board approval (XC16TISI0014K). General information on the three CRC patients is presented in Supplementary Table 1. After preparing frozen blocks of the primary CRC tissues, they were cut and stained with hematoxylin and eosin (H&E). The H&E-stained slides were reviewed by a pathologist to mark tumor cell-rich areas and used as a guide for manual microdissections. Tumor DNA was extracted using the DNeasy blood and tissue kit (Qiagen, Hilden, Germany) and eluted using 50 μL of nuclease-free water. Genomic DNA was also extracted from the blood of the same CRC patients using the same kit. The DNA was quantified with the Qubit dsDNA HS assay kit on a Qubit fluorometer (Thermo Fisher Scientific, Waltham, MA, USA) and stored at -20℃.

### Targeted deep sequencing of the tumor DNA

We performed target deep sequencing of the genomic DNA extracted from the three CRC tissue samples and matched normal samples using a custom NGS panel (OncoChase-AS01, ConnectaGen, Seoul, Korea), targeting 95 cancer-related genes as described elsewhere [10,11]. Tumor DNA was amplified, digested, and barcoded using the Ion Ampliseq library kit 2.0 (Thermo Fisher Scientific) and Ion Xpress barcode adapter kit (Thermo Fisher Scientific) as described elsewhere [10]. The libraries were then templated on an Ion Chef system (Thermo Fisher Scientific) using Ion 520 and Ion 530 Chef reagents (Thermo Fisher Scientific). The prepared libraries were sequenced on an Ion S5 sequencer using an Ion 530 chip and Ion S5 sequencing reagents (Thermo Fisher Scientific) as described elsewhere [10].

### Extraction of cfDNA from patients’ blood

From the three CRC patients, ethylenediaminetetraacetic acid–treated whole blood samples were collected and centrifuged at 2,000 ×g for 10 min at room temperature. The plasma layer was isolated and centrifuged at 16,000 ×g for 10 min to remove contaminated cells. Then, cfDNA was extracted from the plasma using a QIAamp circulating nucleic acid kit (Qiagen) and the DNA was eluted using 50 μL of nuclease-free water. The DNA was quantified using the Qubit dsDNA HS assay kit on a Qubit fluorometer (Thermo Fisher Scientific).

### Digital PCR

We purchased a primer/probe mix targeting three mutations in the *KRAS* gene (p.G12V, p.G12C, and p.G13D; wet lab-validated custom TaqMan SNP genotyping assays, Thermo Fisher Scientific). The details are available in Supplementary Table 2. The dPCR experiments were performed using the QuantStudio 3D digital PCR system (Thermo Fisher Scientific) according to the manufacturer’s instructions. In brief, 14.5 μL of the dPCR reaction mixture was prepared, which contained 7.2 μL of QuantStudio 3D digital PCR master mix v2 (Thermo Fisher Scientific), 1 μL of plasma cfDNA (10 ng/μL), and 0.75 μL of the primer/probe set (final concentrations of 900 nM/250 nM, respectively). The reaction mixture was loaded onto a QuantStudio 3D digital PCR 20K chip v2 (Thermo Fisher Scientific) and run on a ProFlex PCR system (Thermo Fisher Scientific) under the following program: 96℃ for 10 min, 39 cycles at 60℃ for 2 min, 98℃ for 30 s, and 60℃ for 2 min. After amplification, fluorescence signals were analyzed using the QuantStudio 3D digital PCR software v3.0.

## Results

### Genetic alteration profiles of the three CRC tissue samples

We examined the genetic alteration profiles of the three CRC tissue samples by targeted NGS with an OncoChase-AS01 panel covering 95 well-known cancer genes, as described in the Materials and Methods section. Blood DNA from the same CRC patients was also sequenced as a matched normal control to determine which somatic alterations were present. The average coverage of the sequencing depth was 1,319× (range, 976.9× to 1,946×). Through the targeted NGS analysis, we identified 11 non-silent mutations across six cancer-related genes (*APC, KRAS, TP53, TERT, ARIDIA*, and *BRCA1*) (Table 1). Of the six mutations, four (*KRAS, TP53, APC*, and *ARID1A*) are listed in the top 20 CRC genes in the COSMIC database (http://cancer.sanger.ac.uk/cosmic).

### Detection of *KRAS* mutations by dPCR and comparison of the results with targeted NGS

**Fig. 1.**
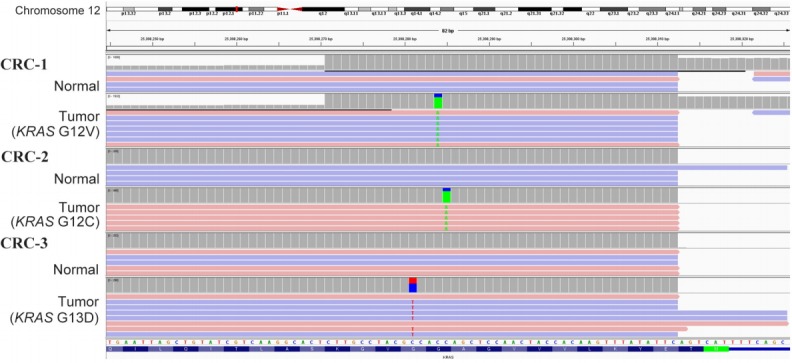
Identification of somatic *KRAS* mutations by targeted next-generation sequencing from a set of three colorectal cancer (CRC) samples. The green letter A and red letter T indicate the presence of a non-reference allele. Somatic point mutations of C>A at 25,398,284 bp for CRC-1, C>A at 25,398,285 bp for CRC-2, and C>T at 25,398,281 bp for CRC-3 were found in *KRAS* on chromosome 12.

**Fig. 2.**
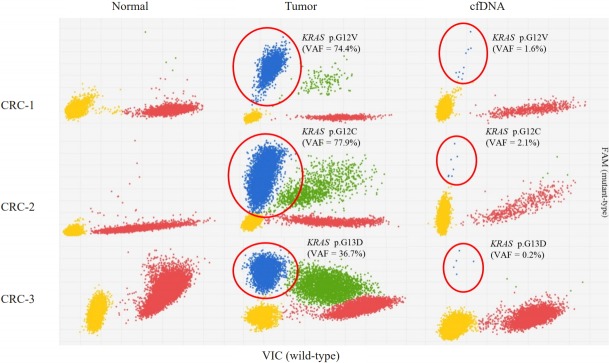
*KRAS* mutations identified in the three colorectal cancer (CRC) tissue samples and plasma cell–free DNA (cfDNA) from the same patients by dPCR. Blue (FAM dye) represents the *KRAS* mutant-type allele and red (VIC dye) represents the wild-type allele. VAF, variant allele frequency.

All three samples had *KRAS* mutations, and all of the *KRAS* mutations were well-known driver events (Table 1, Fig. 1). Therefore, we selected *KRAS* mutations as the target of dPCR. We examined whether dPCR could identify the *KRAS* mutations identified by targeted NGS analysis. When we performed dPCR using the same CRC tissue samples, all expected *KRAS* mutations were clearly identified by dPCR (Table 2, Fig. 2). The variant allele frequencies (VAFs) of the three mutations identified by dPCR were largely consistent with those identified by targeted NGS: CRC-1 (G12V), 74.4% versus 74.2%; CRC-2 (G12C), 77.9% versus 75.8%; and CRC-3 (G13D), 36.7% versus 40.9%, respectively (Table 2).

### Identification of *KRAS* mutations in plasma cfDNA by dPCR

We next applied the dPCR system to analyze the plasma cfDNA isolated from the three CRC patients. All three *KRAS* mutations were consistently identified in the CRC patients, although the VAFs of the three mutations were much lower than those found in the CRC tissue sample: CRC-1 (G12V), 1.613%; CRC-2 (G12C), 2.638%; and CRC-3 (G13D), 0.166% (Table 2, Fig. 2). There was no non-specific identification of *KRAS* mutations.

## Discussion

Recently, liquid biopsy has been proposed as an attractive tool for the non-invasive follow-up of cancer treatment outcomes. However, in terms of robustness and accessibility, the NGS approach is not yet suitable to apply for liquid biopsy samples in the clinical field. In contrast, dPCR can detect mutations sensitively and quantify them without a standard curve [12]. Regarding robustness, accessibility, and sensitivity, dPCR is suitable for screening the mutation profiles of liquid biopsy samples. Despite these advantages, dPCR has limitations in multiplex identification. Considering its advantages and limitations, dPCR may be ideal for screening the key driver mutations in liquid biopsy samples. In this study, we aimed to verify whether the mutations identified in the primary tumor tissue could be consistently detected in plasma cfDNA by dPCR. We first checked whether the mutations identified by the NGS analysis of the primary tumor tissue samples were consistently detected by dPCR. We also compared the VAFs of the mutations identified by NGS and dPCR in the same CRC tissues. Finally, we determined whether the mutations identified in the tumor tissues were detected in plasma cfDNA by dPCR.

For this, we selected *KRAS* mutations because all three CRC patients had *KRAS* mutations, which are among the most important driver mutations in many cancers, including CRC [13,14]. When we used dPCR to examine the *KRAS* mutations that were identified by the NGS analysis, all expected mutations were consistently identified and the minor allele frequencies (MAFs) were almost identical between the NGS and dPCR results. These data provide support for dPCR as a robust and reliable tool for identifying target mutations. When we explored whether the *KRAS* mutations identified in the CRC tumor tissue samples were consistently detected in the plasma cfDNA of the three CRC patients by dPCR, all three *KRAS* mutations were consistently identified. This data suggests that liquid biopsy-based mutation screening may be a useful tool for the non-invasive follow-up of cancer treatment. However, the MAFs of all three mutations were quite low (0.166%–2.638%), which is consistent with previous observations that the MAFs of mutations identified in cfDNA were much lower than those obtained from tumor tissues and were sometimes inconsistent [15]. Although we did not compare the mutation detection performance of dPCR and NGS using cfDNA, mutations with an MAF in that range might not be consistently detectable by NGS. All these data suggest that dPCR may be a sensitive, robust, and cost-effective tool for liquid biopsy-based cancer treatment follow-up.

In conclusion, we confirmed that the *KRAS* mutations identified from the CRC tumor tissue samples were consistently detected in the plasma cfDNA of the three CRC patients by dPCR. Our data suggest that dPCR may be a suitable tool for liquid biopsy-based precision medicine.

## Figures and Tables

**Table 1.** Mutations in three colorectal cancer samples using the OncoChase cancer panel

Sample ID	Chr	Position^a^	Gene	AA change	VAF (%)	COSMIC^b^ ID
CRC-1	5	112,128,191	*APC*	R232X	74.2	COSM13130
	12	25,398,284	*KRAS*	G12V	74.2	COSM520
	17	7,577,559	*TP53*	C242Afs*5	61.1	COSM44657
CRC-2	5	1,293,859	*TERT*	R381H	20	COSM5773048
	12	25,398,285	*KRAS*	G12C	75.8	COSM516
	17	7,578,406	*TP53*	R175H	73.3	COSM10648
CRC-3	5	112,128,143	*APC*	R216X	57.7	COSM98420
	1	27,107,096	*ARID1A*	R2236H	22.6	-
	17	41,226,482	*BRCA1*	S1535F	18.5	-
	12	25,398,281	*KRAS*	G13D	40.9	COSM532
	17	7,577,538	*TP53*	R248Q	46.7	COSM10662

Chr, chromosome; AA, amino acid; VAF, variant allele frequency.

a Genomic position was mapped to hg19;

b COSMIC (Catalogue of Somatic Mutations in Cancer, http://cancer.sanger.ac.uk/cosmic).

**Table 2.** Summary of 3D digital PCR data

					OncoChase	Digital PCR	
Gene	AA change	Sample ID	Sample type		VAF (%)	VAF (%)	CI (%)
*KRAS*	G12V	CRC-1	Tumor		74.2	74.4	70.3–78.8
			cfDNA		-	1.6	0.9–3.0
*KRAS*	G12C	CRC-2	Tumor		75.8	77.9	74.6–81.3
			cfDNA		-	2.1	1.2–3.9
*KRAS*	G13D	CRC-3	Tumor		40.9	36.7	35.1–38.5
			cfDNA		-	0.2	8.6×10^-2^–0.3

PCR, polymerase chain reaction; AA, amino acid; VAF, variant allele frequency; CI, confidence interval; cfDNA, plasma cell–free DNA.

## References

[1] Swanton C (2012). Intratumor heterogeneity: evolution through space and time. Cancer Res.

[2] Siravegna G, Marsoni S, Siena S, Bardelli A (2017). Integrating liquid biopsies into the management of cancer. Nat Rev Clin Oncol.

[3] Crowley E, Di Nicolantonio F, Loupakis F, Bardelli A (2013). Liquid biopsy: monitoring cancer-genetics in the blood. Nat Rev Clin Oncol.

[4] Diaz LA, Bardelli A (2014). Liquid biopsies: genotyping circulating tumor DNA. J Clin Oncol.

[5] Beck J, Bierau S, Balzer S, Andag R, Kanzow P, Schmitz J (2013). Digital droplet PCR for rapid quantification of donor DNA in the circulation of transplant recipients as a potential universal biomarker of graft injury. Clin Chem.

[6] Devonshire AS, O'Sullivan DM, Honeyborne I, Jones G, Karczmarczyk M, Pavsic J (2016). The use of digital PCR to improve the application of quantitative molecular diagnostic methods for tuberculosis. BMC Infect Dis.

[7] Thress KS, Brant R, Carr TH, Dearden S, Jenkins S, Brown H (2015). *EGFR* mutation detection in ctDNA from NSCLC patient plasma: a cross-platform comparison of leading technologies to support the clinical development of AZD9291. Lung Cancer.

[8] Burjanivova T, Malicherova B, Grendar M, Minarikova E, Dusenka R, Vanova B (2019). Detection of *BRAF*V600E mutation in melanoma patients by digital PCR of circulating DNA. Genet Test Mol Biomarkers.

[9] Garcia-Murillas I, Lambros M, Turner NC (2013). Determination of *HER2* amplification status on tumour DNA by digital PCR. PLoS One.

[10] Yu SM, Jung SH, Chung YJ (2018). Comparison of the genetic alterations between primary colorectal cancers and their corresponding patient-derived xenograft tissues. Genomics Inform.

[11] Choi SH, Jung SH, Chung YJ (2017). Validation of customized cancer panel for detecting somatic mutations and copy number alterations. Genomics Inform.

[12] Day E, Dear PH, McCaughan F (2013). Digital PCR strategies in the development and analysis of molecular biomarkers for personalized medicine. Methods.

[13] Perincheri S, Hui P (2015). *KRAS* mutation testing in clinical practice. Expert Rev Mol Diagn.

[14] Zhuang R, Li S, Li Q, Guo X, Shen F, Sun H (2017). The prognostic value of *KRAS* mutation by cell-free DNA in cancer patients: a systematic review and meta-analysis. PLoS One.

[15] Christensen E, Nordentoft I, Vang S, Birkenkamp-Demtroder K, Jensen JB, Agerbaek M (2018). Optimized targeted sequencing of cell-free plasma DNA from bladder cancer patients. Sci Rep.

