# Supplementary material for: Detection of KRAS mutations in plasma cell-free DNA of colorectal cancer patients and comparison with cancer panel data for tissue samples of the same cancers
Source: Genomics Inform. 2019 Nov 29;17(4):e42. doi: 10.5808/GI.2019.17.4.e42 (PMC6944046; doi:10.5808/GI.2019.17.4.e42)
Supplement: Supplementary file 1 [file gi-2019-17-4-e42-supplementary.pdf]

Supplementary Table 1. Clinical information on patients' primary cancers

Sample ID	Age (yr)	Sex	Diagnosis	Pathology
CRC-1	81	Male	Colorectal cancer	Adenocarcinoma
CRC-2	49	Male	Colorectal cancer, lung metastasis	Adenocarcinoma
CRC-3	66	Male	Colorectal cancer, lung metastasis	Adenocarcinoma

Supplementary Table 2. Detailed information on the primer/probe mix used for digital PCR

Assay ID	Gene	AA change	Nucleotide change	Wild-type allele	Mutant-type allele
AHX1IHY	*KRAS*	G12V	c.35G>T	C	A
AH0JEUD	*KRAS*	G12C	c.34G>T	G	T
AHD2BW0	*KRAS*	G13D	c.38G>A	C	T

AA, amino acid; PCR, polymerase chain reaction.
